# Computed Tomography Perfusion Imaging Detection of Microcirculatory Dysfunction in Small Intestinal Ischemia-Reperfusion Injury in a Porcine Model

**DOI:** 10.1371/journal.pone.0160102

**Published:** 2016-07-26

**Authors:** Haifeng Shi, Ruokun Li, Jinwei Qiang, Ying Li, Li Wang, Rongxun Sun

**Affiliations:** 1 Department of Radiology, Changzhou NO2 People's Hospital, Changzhou, Jiangsu 213000, China; 2 Department of Radiology, Jinshan Hospital, Shanghai Medical College, Fudan University, Shanghai 201508, China; 3 Department of Pathology, Jinshan Hospital, Shanghai Medical College, Fudan University, Shanghai 201508, China; 4 Department of Surgery, Jinshan Hospital, Shanghai Medical College, Fudan University, Shanghai 201508, China; Duke University Medical Center, UNITED STATES

## Abstract

**Objective:**

To evaluate multi-slice computed tomography (CT) perfusion imaging (CTPI) for identifying microcirculatory dysfunction in small intestinal ischemia−reperfusion (IR) injury in a porcine model.

**Materials and Methods:**

Fifty-two pigs were randomly divided into 4 groups: (1) the IR group (n = 24), where intestinal ischemia was induced by separating and clamping the superior mesenteric artery (SMA) for 2 h, followed by reperfusion for 1, 2, 3, and 4 h (IR-1h, IR-2h, IR-3h, and IR-4h; n = 6, respectively); (2) the sham-operated (SO) group (n = 20), where the SMA was separated without clamping and controlled at postoperative 3, 4, 5, and 6 h (SO-3h, SO-4h, SO-5h, and SO-6h; n = 5, respectively); (3) the ischemia group (n = 4), where the SMA was separated and clamped for 2 h, without reperfusion, and (4) baseline group (n = 4), an additional group that was not manipulated. Small intestinal CTPI was performed at corresponding time points and perfusion parameters were obtained. The distal ileum was resected to measure the concentrations of malondialdehyde (MDA) and superoxide dismutase (SOD) and for histopathological examination.

**Results:**

The perfusion parameters of the IR groups showed significant differences compared with the corresponding SO groups and the baseline group (before ischemia). The blood flow (BF), blood volume (BV), and permeability surface (PS) among the 4 IR groups were significantly different. BF and BV were significantly negatively correlated with MDA, and significantly positively correlated with SOD in the IR groups. Histopathologically, the effects of the 2-h ischemic loops were not significantly exacerbated by reperfusion.

**Conclusion:**

CTPI can be a valuable tool for detecting microcirculatory dysfunction and for dynamic monitoring of small intestinal IR injury.

## Introduction

The intestine is probably the most sensitive organ to ischemia−reperfusion (IR) injury [1], a phenomenon that occurs in many disorders, such as strangulated hernia, volvulus, necrotizing enterocolitis, mesenteric embolism, procoagulant activation, and septic and hypovolemic shock [2]. A key consequence of intestinal IR injury is breakdown of the intestinal barrier function, which results in bacterial translocation, endotoxemia, and uncontrolled release of inflammatory mediators and cytokines; this induces systemic inflammatory response syndrome (SIRS) and injury of distant organs [3, 4]. Multiple organ dysfunction syndrome (MODS) or multiple organ failure (MOF) may also occur [5]. Dysfunction of the microcirculation is considered to be a major determinant of the development and deterioration of intestinal IR injury and precedes the development of tissue injury and gut failure [6]. Thus, it is crucial to detect microcirculation dysfunction prior to the development of intestinal tissue injury.

Several techniques, such as laser Doppler velocimetry, tissue reflectance spectrophotometry, and intravital fluorescence microscopy have been used to investigate the intestinal microcirculation [7–9]. However, these techniques are invasive or are not clinically available. Computed tomography (CT) perfusion imaging (CTPI) has become a useful tool for the evaluation of tissue microcirculation in clinical practice [10, 11]. It has been used to assess vascular pathophysiology noninvasively and to measure tissue perfusion in the brain, kidney, heart, spleen, and pancreas [12, 13].

We hypothesized that CTPI could be used to evaluate intestinal IR injury. Thus, the purpose of this study was to investigate the usefulness of CTPI for the detection of microcirculatory dysfunction in intestinal IR injury, and to correlate these findings with clinical laboratory markers.

## Materials and Methods

### Animals and experimental procedures

This study was approved by the institutional review board of Jinshan hospital, Shanghai Medical College, Fudan University. Fifty-twoBama pigs weighting 20−25 kg (22.9 ± 1.4 kg), with ages ranging from 130 to 150 days (License SCXK 2010–0025, Shanghai Academy of Agricultural Sciences, Shanghai, China) were acclimated and housed at the animal care center for 1 week. After fasting for 24 h, the animals were anesthetized with an intramuscular injection of ketamine (10 mg/kg bodyweight) and atropine (0.02 mg/kg bodyweight) in the thigh; anesthesia was maintained by sevoflurane inhalation via endotracheal intubation. The right femoral artery was cannulated and connected to a transducer for continuous monitoring of the blood pressures. The blood pressures were recorded at every studied time point and mean arterial pressure(MAP) was calculated. The respiratory rate, pulse rate, and oxygen saturation were also monitored.

All surgical manipulations were performed by an experienced general surgeon (R.X.S.) under aseptic conditions. Fifty-two pigs were randomly assigned to the IR, sham-operated (SO), ischemia, and baseline groups. A midline laparotomy was performed, and the entire small intestine was visualized. In the IR group (24 pigs), ischemia was induced by separating and clamping the superior mesenteric artery (SMA) for 2 h, followed reperfusion induced by declamping the SMA for 1 h, 2 h, 3 h, or 4 h (IR-1h, IR-2h, IR-3h, and IR-4h; n = 6, respectively). The SO groups (20 pigs) served as controls; in these pigs, the SMA was separated without clamping and controlled at postoperative 3, 4, 5, and 6 h (SO-3h, SO-4h, SO-5h and SO-6h; n = 5, respectively) for comparison with the corresponding IR-1h, IR-2h, IR-3h, and IR-4h groups. In the ischemia group (n = 4), the SMA was separated and clamped for 2 h, without reperfusion. The baseline group (n = 4) were non-manipulated pigs and was used to obtain normal values.

### CTPI

All animals underwent CTPI using a 128-slice CT scanner (Siemens, Erlangen, Germany). The abdominal belt was used to minimize motion artifacts. Prior to CTPI, an unenhanced scan of the entire abdomen was performed to identify the small intestine. CTPI was performed at the baseline (before SMA clamping), in the 4 IR groups, 4 SO groups, the ischemia group,in a continuous volume scan pattern at 3.5-s intervals using the "abdomen VPCT long mode." The scan range extended from the middle to the lower abdomen, which included the majority of the small intestine, with a coverage of 16.8 cm. The scan began at 5 s after an intravenous injection of 50 ml of non-ionic iodinated contrast medium (iopromide, 370 mg I/ml; Bayer Healthcare, Berlin, Germany) at a rate of 5 ml/s using a power injector, followed by a saline flush (injection of 40 ml of saline). Acquisition lasted for 54.08 s with parameters of 80 kV, 110 mA, and 6 mm collimation.

### Image analysis

The raw images were post-processed on a workstation (Syngo, Siemens, Berlin, Germany) using a VPCT body-perfusion model. As proposed by Khan et al.[14], the arterial input was localized in the lumen of the abdominal aorta. Color maps of CT perfusion parameters were generated according to the maximum slope method. After correction for motion, a distal ileum loop, which was perpendicular to the transverse plane, was selected for quantitative analysis. All analyses were performed by 2 experienced radiologists (H.F.S. and R.K.L.) as follows: (a) the image was magnified to double the original size; (b) an ellipsoid region of interest (ROI), which ranged from 10 to 30 pixels (mean: 20 pixels), was placed in the small bowel and care was taken to avoid the surrounding fat tissue, intraluminal gas, or motion artifacts; (c) CT perfusion parameters, including blood flow (BF), blood volume (BV), mean transit time (MTT), and permeability surface (PS), were measured 6 times (3 times per radiologist) in different ileum segments, and the values were averaged in all animals except the ischemia group (perfusion parameterscan not be measured) which were only used for histopathology purposes.

### Histopathology

After CT examination, all animals were sacrificed with an intravenous injection of 10% KCl. A 3-cm distal ileum segment (20 cm distal from the ileocecal valve) was resected for evaluation of intestinal wall injury according to Chiu’s morphological standards [15]. Criteria of Chiu grading system consists from 5 subdivisions according to the changes of villus and gland of intestinal mucosa: grade 0, normal mucosal villi; grade 1, development of subepithelial space, usually at tip of villus,with capillary congestion; grade 2, extension of subepithelial space with moderate lifting of epithelial layer; grade 3, massive epithelial lifting down sides of villi; grade 4, denuded villi with lamina propria and dilated capillaries exposed; grade 5, digestion and disintegration of lamina propria, hemorrhage and ulceration. The pathological specimens were fixed in 10% formalin solution, embedded in paraffin, cut into 5-μm sections with a microtome, and stained with hematoxylin and eosin (HE). Three sections were obtained from the same bowel segment and examined under a light microscope by an experienced pathologist (L.W.) in a blinded fashion.The mucosal tissues of a 20-cm distal ileum segment were scraped with a glass slide and frozen in liquid nitrogen for subsequent biochemical analysis.

### Biochemical analysis

A distal ileum segmentwas resected for evaluating Malondialdehyde (MDA) levels and Superoxide dismutase (SOD) activity. MDA levels were measured according to the method previously described by Wonget al. [16]. SOD activity was assayed using the nitro blue tetrazolium method, as previously described by Sunet al. [17].

### Statistical analysis

Statistical analyses were performed using the SPSS 16.0 package (SPSS Inc., Chicago, IL) for Windows and with Microsoft Excel 2007 (Microsoft Corporation, Redmond, WA). Data of perfusion parameters were expressed as median (range). Data of MAP, biochemical markers, and histological grades were expressed as mean ± SD. One-way ANOVA analysis and the Student−Newman−Keuls test were used for multiple comparisons of data among the baseline, SO, ischemia, and IR groups, in the following categories: MAP, biochemical markers, and histological grades. Chi-square test was used to compare the difference in perfusion parameters. Correlations between the perfusion parameters and MDA and SOD were analyzed using Spearman’s correlation coefficient analysis. A p-value of less than 0.05 was considered to be statistically significant.

## Results

Two animals died in the IR-2h and IR-4h groups, after reperfusion of 1.5 h and 3.6 h, respectively, and thus were excluded from further analysis. Perfusion did not occur after clamping in the ischemia group, and hence comparison of perfusion parameters between the IR groups and ischemia group was not performed.Perfusion parameters, biochemical markers, and histological grades in the different groups are listed in Table 1.

**Table 1:** Perfusion parameters, biochemical markers and histological grades in the different groups(median or mean ± SD).

Time	BF	BV	MTT	PS	MDA	SOD	Histological
	(ml/100 ml/min)	(ml/100 ml)	(s)	(ml/100 ml/min)	(nmol/mg prot)	(U/mg prot)	grade
Baseline	30.55(24.26–44.58)	8.77(8.12–11.99)	19.51(16.23–28.74)	23.77(17.16–31.5)	0.81 ± 0.08	22.99 ± 2.56	0.60 ± 0.54
IR-1h	35.625(27–56.11)	7.375(5.75–11.08)	10.12(9.12–13.56)^⇞^*	19.75(12.5–24.33)	0.87 ± 0.39	20.72 ± 6.17	3.17 ± 1.17^⇞^*
SO-3h	24.25(19.5–40)	4.985(3.75–6.32)	10.395(9.3–14.47)	12.375(9–17)	0.84 ± 0.93	21.71 ± 1.81	0.75 ± 0.50
IR-2h	22.5(19.5–32.25)*^↑^	4.27(4.1–5.72)^⇞^*	12(9.7–14.2)^⇞^*	14.83(12–21.5)	1.22 ± 0.13	14.04 ± 2.94	3.67 ± 1.10^⇞^*
SO-4h	29(19.48–39.03)	8.62(8.12–12.35)	18.79(16.58–21.64)	19.86(18.61–29.34)	0.76 ± 0.11	22.21 ± 1.09	0.67 ± 0.57
IR-3h	24.25(19.5–40)^↑^	4.985(3.75–6.32)^⇞^*	10.395(9.3–14.47)^⇞^*	12.375(9–17)^⇞^*	1.00 ± 0.49	19.21 ± 6.14	3.51 ± 1.16^⇞^*
SO-5h	32.13(27.5–44.58)	8.62(8.12–11.61)	18.87(16.23–28.74)	24.91(19.13–31.5)	0.82 ± 0.27	22.98 ± 3.61	0.75 ± 0.50
IR-4h	17.75(14.5–25.75)^↑^*^⇞^	3.55(2.44–4.33)^↑^^⇞^*	13.22(8.6–15.27)^⇞^*	11.5(9–12.6)^⇞^*	1.67 ± 0.55*^↑^^⇞^	10.53 ± 1.95*^↑^^⇞^	3.61 ± 1.16^⇞^*
SO-6h	29.45(24.26–43.87)	8.77(8.46–10.33)	17.68(17.55–27.28)	24.17(22.89–27.97)	0.79 ± 0.19	21.71 ± 1.81	0.80 ± 0.44

p< 0.05 vs. baseline group (*), corresponding SO group (⇞), and IR-1h group (↑).

### Comparison of perfusion parameters between the IR and SO groups

**Fig 1:**
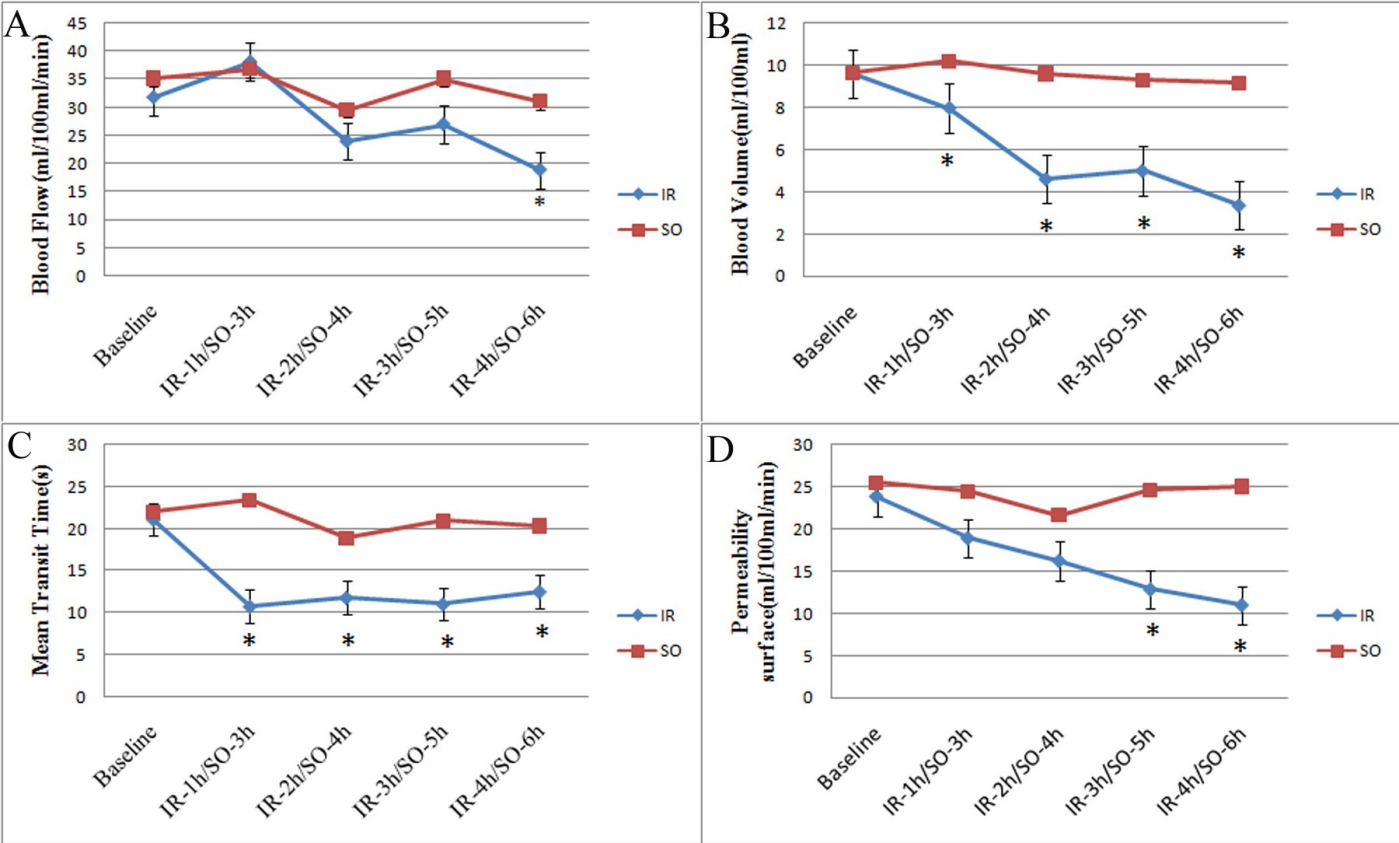
Perfusion parameters in ischemia-reperfusion(IR) animals and corresponding sham-operated(SO) animals. (A) BF in IR animals and corresponding SO animals. (B) BV in IR animals and corresponding SO animals. (C) MTT in IR animals and corresponding SO animals. (D) PS in IR animals and corresponding SO animals.Data are presented as median (n = 24 pigs in the IR group and n = 20 in the SO group:*p < 0.05VS time-matched control)

Perfusion parameters showed no significant differences between the SO groups and the baseline group, or among the 4 SO groups (all p > 0.05). The perfusion parameters between the IR groups and the SO groups differed significantly. The BF value of the IR-4h was significantly lower than that in the corresponding SO-6h group (p = 0.018). The BV values of the IR-2h, IR-3h, and IR-4h groups were significantly lower than those in the corresponding SO-4h, SO-5h, and SO-6h groups (p = 0.003, p < 0.001, p < 0.001, respectively). The MTT values of the 4 IR groups were significantly lower than those in the corresponding 4 SO groups (p < 0.001, p < 0.001, p = 0.011, and p = 0.008, respectively). The PS values of the IR-3h and IR-4h groups were significantly lower than those in the corresponding SO groups (p = 0.001, p < 0.001, respectively; Fig 1).

#### Comparison of perfusion parameters among IR groups

Perfusion parameters are summarized in Table 1 and Fig 1. The BF, BV, and PS values among the 4 IR groups were significantly different (p = 0.005, p < 0.001, and p = 0.007, respectively). The MTT values among the 4 IR groups showed no significant difference (p = 0.191). The BF value did not decrease in the IR-1h group, but were significantly decreased in the IR-2h and IR-4h groups as compared with the baseline group (p = 0.017 and p = 0.001, respectively). The BF values significantly decreased in the IR-2h, IR-3h, and IR-4h groups as compared with the IR-1h group (p = 0.008, p = 0.025, p = 0.001, respectively). BF values did not differ significantly among the IR-2h, IR-3h, and IR-4h groups (all p > 0.05). The BV values were slightly decreased in the IR-1h group (p = 0.183), and significantly decreased in the IR-2h, IR-3h, and IR-4h group as compared with the baseline group (all p < 0.001), and there was a significant difference between the IR-1h and the IR-4h group (p = 0.014). There were no significant differences among the IR-2h, IR-3h, and IR-4h groups (all p > 0.05). Compared with the baseline group, the MTT values were significantly decreased inthe IR-1h,IR-2h, IR-3h, and IR-4h groups (all p < 0.001). There were no significant differences among the 4 reperfusion time points (all p > 0.05). The PS values progressively decreased with reperfusion time, with a significant difference between the IR-1h, IR-2h, IR-3h, and IR-4h groups and the baseline group (p = 0.0018, p = 0.001, p < 0.001, and p < 0.001, respectively), and in the IR-1h compared with the IR-3h and IR-4h groups (p = 0.008 and p = 0.002, respectively).

### Data of MAP

**Fig 2:**
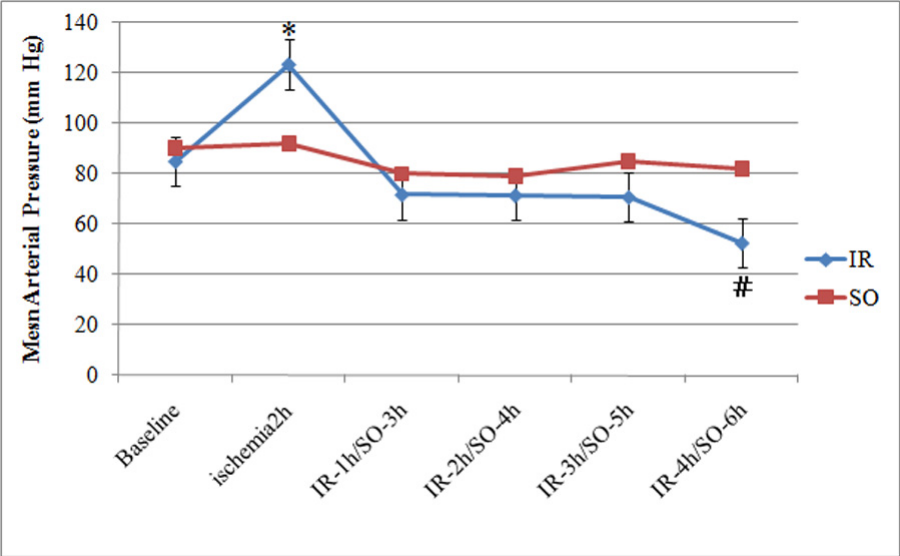
Mean arterial pressure measurements in IR animals and corresponding SO animals. *p < 0.001 vs. baseline, IR and SO groups.#p < 0.001 vs. baseline, and corresponding SO groups.)

Similar baseline values in MAP (IR, 85±5.61 vs. SO, 90±6.21mmHg; p > 0.05) were observed in both groups. The time course of MAP changes in animals subjected to IR and respective controls are depicted in Fig 2. MAP in SO animals was stable over the study period. However, in IR groups, mesenteric occlusion resulted in a pronounced increase in MAP in comparison to other groups (Fig 2; p< 0.001). After the reperfusion of the SMA, the decrease of MAP was more pronounced and abrupt in the IR-4h groups compared with the baseline and corresponding SO groups(Fig 2; p< 0.001).

### Biochemical markers

**Fig 3:**
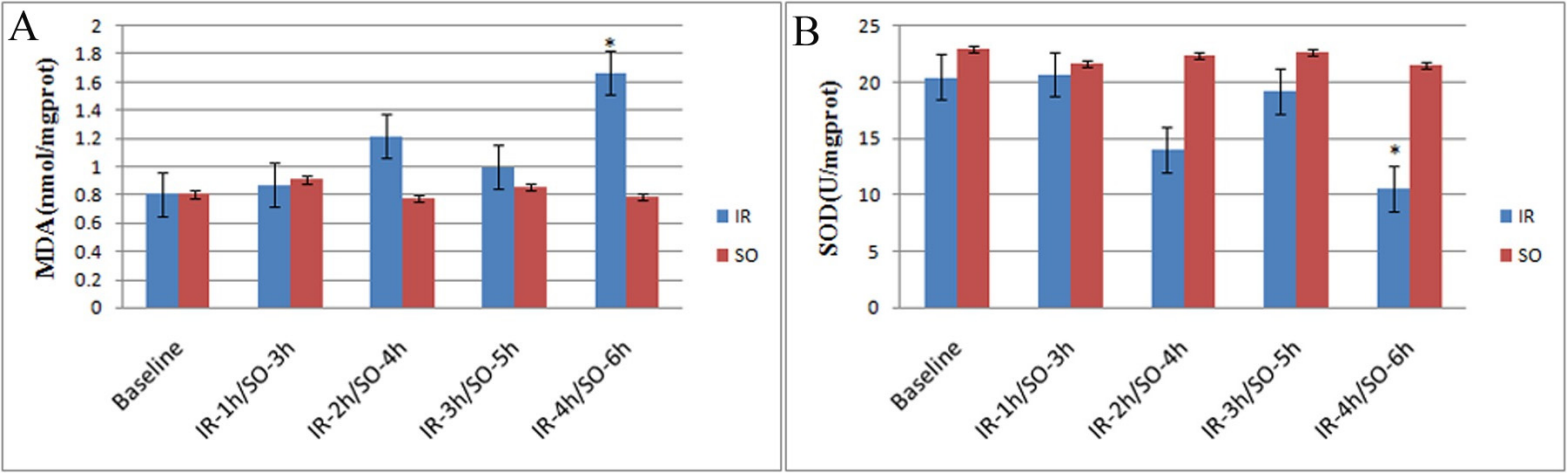
The variation of Malondialdehyde (MDA) and Superoxide dismutase (SOD) originated from intestinal mucosa homogenate. An increasing trend in MDA levels(A) and a decreasing trend in SOD (B) activity with the increase in IR time can be observed; however, some regression is observed in the IR-3h group.*p < 0.01 vs. corresponding SO groups.)

**Fig 4:**
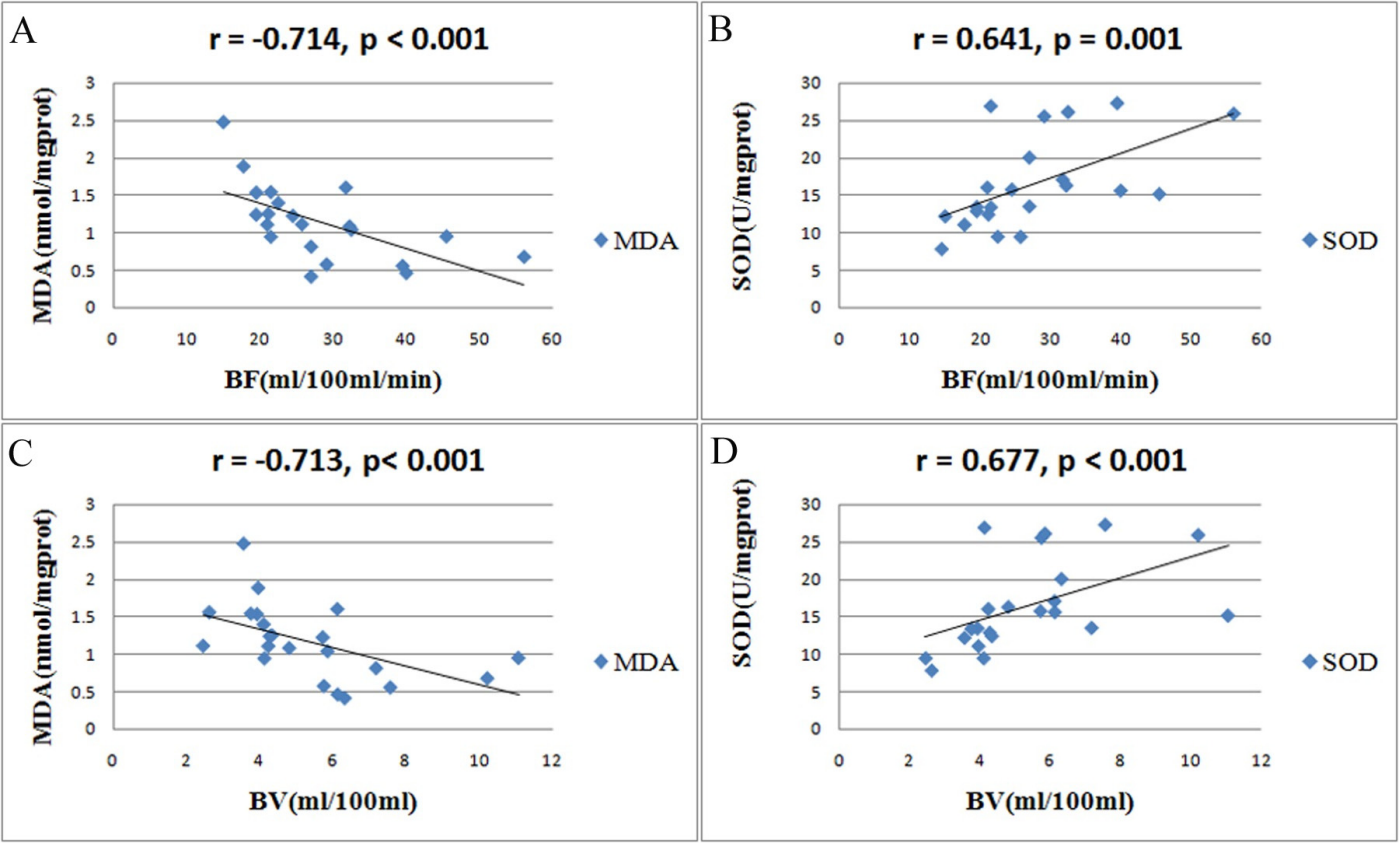
Correlations between the perfusion parameters and biochemical markers. (A) correlation between BF and MDA. (B) correlation between BF and SOD. (C) correlation between BV and MDA. (D) correlation between BV and SOD. Scatter plots reveal a negative correlation between BF and BV values and MDA levels, a positive correlation between BF and BV values and SOD activity.)

No difference was observed in MDA and SOD values between the SO groups and the baseline group, or among the 4 SO groups (all p > 0.05). As compared with the baseline group, the MDA value did not increase in the IR-1h group (p = 0.337), was slightly increased in the IR-2h group (p = 0.192), slightly decreased in the IR-3h group (p = 0.611), and significantly increased in the IR-4h (p = 0.006). The SOD value was significantly decreased in the IR-2h group (p = 0.036), slightly increased in the IR-3h group (p = 0.399), and significantly decreased in the IR-4h group (p = 0.003) as compared to baseline values (Fig 3, Table 1). The scatter plots showed a significant negative correlation between the BF and BV values and MDA levels (r = -0.714, p < 0.001; r = -0.713, p< 0.001; respectively), and a significant positive correlation between the BF and BV values and SOD activity (r = 0.641, p = 0.001; r = 0.677, p < 0.001; respectively; Fig 4).

### Histopathology

**Fig 5:**
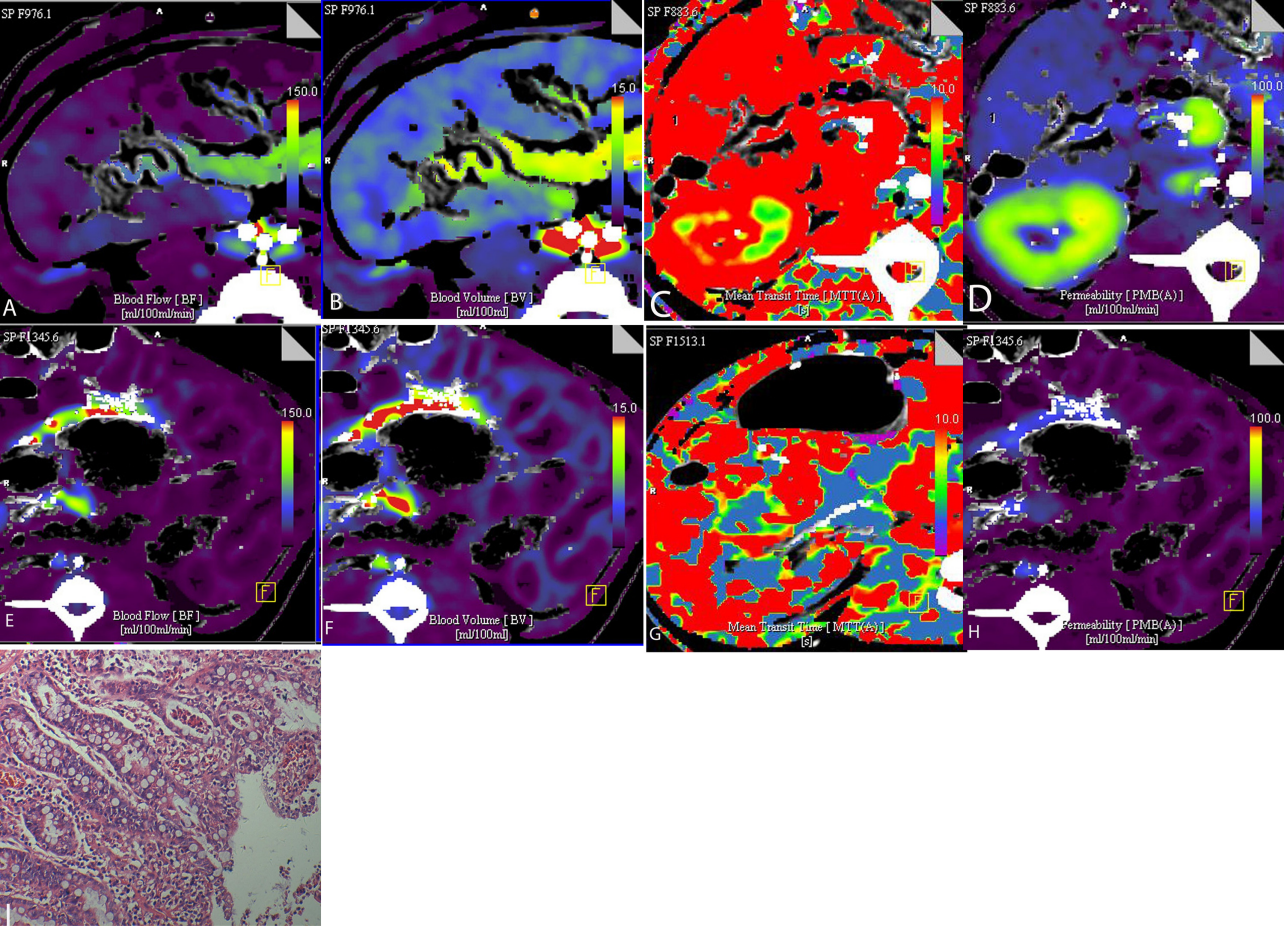
Color maps of CT perfusion parameters. The BF value (E), BV value (F), MTT (G) and PS (H) maps for reperfusion of 4 h reveal that the BF, BV, and PS values are significantly decreased as compared to SO group(A-D). Microscopic ileal histology images show epithelial clefting, villi denudation (Chiu’s score 4) in IR groups (I, HE × 100).)

**Fig 6:**
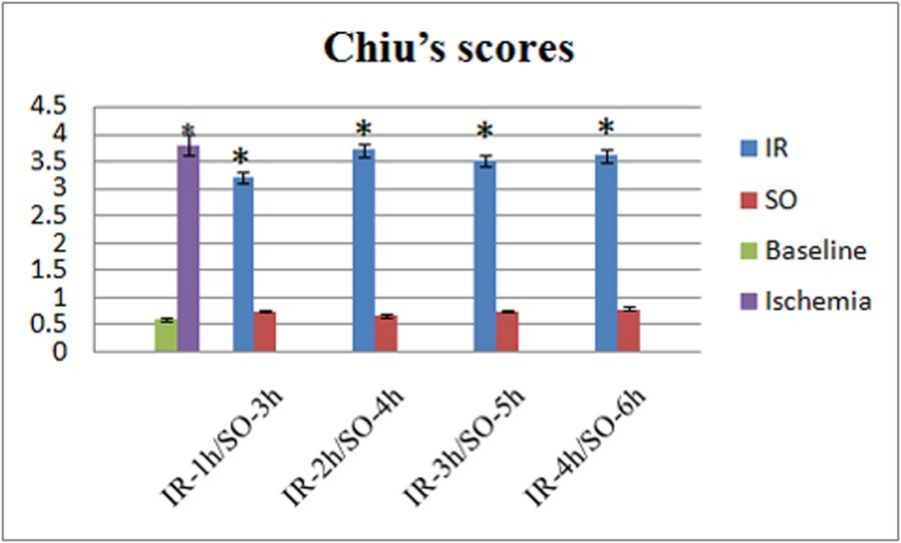
The Chiu’s scores of intestinal villus in different groups. *p < 0.01VS baseline and SO groups.)

The baseline and SO groups showed normal intestinal histopathology. The IR groups showed various degrees of subepithelial clefting, as well as frequent villus denudation. Extracellular edema was present along the entire length of the villi and throughout the intestinal layers (Fig 5I). Chiu’s scores differed significantly between the 4 IR groups, the ischemia group and the baseline group (all p < 0.01). There were no significant differences in Chiu’s scores between the IR groups and the ischemia group, and among the 4 IR groups (all p > 0.05; Table 1, Fig 6).

## Discussion

The mechanisms underlying intestinal IR injury are complex, and multiple factors are involved in the pathophysiological process. Owing to the breakdown of the intestinal barrier function, bacterial translocation, and release of inflammatory cytokines, reperfusion does not ameliorate, but rather exacerbates the intestinal function and systemic condition of the individual [18, 19]. As a crucial factor in IR injury, microcirculatory dysfunction is an abnormality in microcirculatory flow, volume, and permeability, which canbe detected using CTPI. Currently, swines present an ideal model for human intestinal research, due to the similarity in size, physiology, and immunology between pigs and humans [19].

CTPI is a perfusion analysis method that offers a wide array of clinical and research applications. Quantitative maps of tissue perfusion can be created from cine CT data, which are displayed on a color scale, which in turn allows quantification of perfusion in absolute units at a high spatial resolution [12]. Sitek et al. [20] have reported that, following motion correction, CTPI of the normal small bowel is feasible using a single-compartment kinetic model. A CTPI study performed by Khan et al. [14] previously demonstrated that the proximal and distal parts of the colon differ in perfusion, which is consistent with their embryological, anatomical, and physiological differences.

The present study showed that the capillary flow in the intestine decreased after IR injury. After 2 h of ischemia, followed by declamping of the SMA, BF was restored in the IR-1h group, but significantly decreased in IR-2h to IR-4h groups; however, there was some recovery in the IR-3h group. Guan et al. [8] found that, after 50 min of ischemia, BF in some villi recovered slowly and reached only 30% of normal values in mice after 50 min of reperfusion. However, other villi did not show any restoration of BF throughout the reperfusion period. IR-induced microcirculatory damage result in declined BF values during the reperfusion period[21].BV followed the same pattern as BF during the reperfusion period. Endothelial cell swelling, vasomotor dysfunction, and capillary narrowing due to an edema-associated increase in interstitial pressure cause decreased BV values [6]. Interestingly, the BF and BV values showed a transient increase during 2−3 h of reperfusion, and the biochemical markers, MDA and SOD, simultaneously decreased and improved, respectively. Thereafter, however, the BF and BV values showed an irreversible decrease and the biochemical markers simultaneously worsened. The underlying mechanisms require further study.

Our study showed no significant differences in MTT values among the 4 IR groups; however, the MTT values were significantly lower in the IR groups compared withthe corresponding SO groups. In our study, a marked and progressive decrease in PS values was observed during the reperfusion period, which was consistent with the findings of previous studies [22, 23]. It is known that intestinal IR can affect not only the intestine, but also distant organs, thereby resulting in systemic sequelae, such as acidosis, sepsis, circulatory shock, MODS, or MOF [3, 24]. The severe shock that occurred at the end of 3 h of reperfusion in our study decreased the intestinal perfusion pressure and, consequently, the PS values.

Our results also demonstrated that induction of intestinal ischemia resulted in a pronounced rise in MAP, which were similar to the studies of Douzinas et al.[3], Khanna et al.[25] and Hayward et al. [26]. Intestinal ischemia can lead to abrupt rise in MAP via a decrease in the baroreceptor input to the medullary vasomotor center in response to reduced splanchnic perfusion[25]. An abrupt decrease in MAP was found at the end of 3h of reperfusion, indicating the severe circulatory shock induced by the intestinal IR injury,which was reported by Douzinas et al.[3]. It could explain that the precipitous decrease of BF, BV and PS values in the IR-4h group.

A key factor that induces intestinal IR injury is the generation of free radicals from oxygen molecules. Under physiological conditions, the damaging effects of free radicals are prevented by SOD, which is an oxidoreductase that catalyzes superoxide anions and hydrogen to yield molecular oxygen and hydrogen peroxide. During IR, excessive SOD is consumed and the natural defense is compromised [27]. MDA is an end-product of lipid peroxidation and is also formed as a product of the cyclooxygenase reaction in prostaglandin metabolism. It can be determined in both tissue and blood and its concentration is directly proportional to the cell damage caused by free radicals [2]. Our study showed that there was a significant negative correlation between the BF and BV values and MDA levels, and a strong positive correlation between the BF and BV values and SOD activity, and thus there is a good correlation between the CT perfusion parameters and the damaging effects of IR.

This study showed that the mucosal injury of ischemic loops was not significantly exacerbated by reperfusion, which was consistent with the study performed by Blikslager et al. [28]. However, other studies have demonstrated exacerbated mucosal injury during reperfusion. In these studies [8, 29], rats or cats were used for IR injury. The levels of xanthine oxidase-xanthine dehydrogenase were much higher in these species compared to pig [19, 28], which may account for the discrepant results in our study. Humans with similar levels of xanthine oxidase-xanthine dehydrogenase as pig enable an extrapolation of this result to humans[28]. Not only does IR result in damage to the intestinal mucosa but it also results in a loss of mucosal barrier function and subsequent bacterial translocation with severe inflammation and life-threatening infections. Imaging studies should not only focus on the morphological but also functional changes of the intestine during the reperfusion time. Application of a new technique of medical imaging for the detection of the loss of barrier and immune functions of intestine during intestinal IR is of great significance.

Our study had some limitations. First, biochemical markers from ischemia alone pigs were not measured, it is is a huge limitation of our study. Second, number of neutrophils in the intestinal mucosal layer were not semi-quantitative or quantitative assessment. Third, although we used an abdominal belt and motion correction to reduce the artifacts caused by respiratory motion, artifacts caused by intestinal peristalsis were inevitable, which degraded the measurements of the CT perfusion parameters. We did not use anisodamine and glucagon to inhibit intestinal peristalsis, because they can improve mesenteric microcirculation [30] and may disturb the natural course of IR. Fourth, because of the intestinal wall is thin, the ROI needs to be relative small and may have introduced a selection bias. Fifth, the acquisition time used was insufficient for measuring PS values reliably, according to a previous study [31]. Standardization of the procedure will be required to ensure the reliability of data. Finally, a radiation dose is inevitable.

In conclusion, the present study shows that there is a good correlation between CT perfusion parameters and the damaging effects of intestinal IR. Taken together, CTPI is a valuable tool for detecting microcirculatory dysfunction and for dynamic surveillance of intestinal IR injury.
